# Phyllodes tumors of the breast: A comprehensive review of diagnosis, treatment, and follow-up

**DOI:** 10.1016/j.clinsp.2025.100617

**Published:** 2025-03-15

**Authors:** Aline Rocha Donato, Rodrigo Goncalves, Jonathan Yugo Maesaka, Fernando Nalesso Aguiar, José Maria Soares, Carlos Alberto Ruiz, José Roberto Filassi, Edmund Chada Baracat

**Affiliations:** a Discipline of Gynecology, Department of Obstetrics and Gynecology, Faculdade de Medicina da Universidade de São Paulo (FMUSP), São Paulo, SP, Brazil; b Mastology Sector of the Gynecology Division, Instituto de Câncer do Estado de São Paulo, Hospital das Clínicas da Faculdade de Medicina da Universidade de São Paulo (HCFMUSP), São Paulo, SP, Brazil; c Breast Pathology Sector of the Department of Pathological Anatomy, Instituto do Câncer do Estado de São Paulo, Universidade de São Paulo, São Paulo, SP, Brazil

**Keywords:** Phyllodes tumor, Breast, Breast cancer, Fibrocystic breast disease, Breast Diseases, Breast Neoplasms

## Abstract

Phyllodes Tumor (PT) of the breast is a rare type of fibroepithelial neoplasm, classified into benign, borderline, and malignant subtypes. These tumors are known to be locally aggressive and have an inherent potential for Local Recurrence (LR), even after adequate surgical treatment. Establishing strategies to reduce LR has become increasingly important, as 21 % to 36 % of patients experience disease recurrence despite achieving negative surgical margins. Although surgical resection of the lesion is the gold standard treatment for all subtypes of phyllodes tumors, the ideal extent of the surgical margin remains a topic of debate with no consensus in the literature. Adjuvant radiotherapy is suggested as a tool to improve local control of the disease and reduce the recurrence rate; however, published data on this approach are currently conflicting. In this review paper, the authors will provide a detailed analysis of the current evidence on the diagnosis, treatment, follow-up, outcomes, and future perspectives of phyllodes tumors.

## Highlights

• Molecular and IHC markers aid in objective classification and outcome prediction of PT.
• Surgery is the gold standard for PT, but margin extent lacks consensus.
• Local recurrence is key in PT prognosis, with margin status as the main risk factor.
• Adjuvant radiotherapy lowers recurrence in borderline/malignant PTs with close margins.

## Introduction

Phyllodes Tumor (PT) of the breast is a rare type of fibroepithelial neoplasm,^1^ classified into benign, borderline, and malignant subtypes.^1^, ^2^, ^3^, ^4^ It occurs more frequently in 35- to 55-year-old women,^1^^,^^2^^,^^5^ and its etiology has not yet been elucidated.^1^

The high Local Recurrence (LR) rate is the most important prognostic factor in these tumors.^2^ The main predictors of recurrence are positive margins and tumor size.^6^ Additionally, the borderline and malignant subtypes tend to grow fast and may develop distant metastases.^2^

Surgical resection of the lesion is the gold standard treatment,^1^ despite the lack of consensus about the ideal surgical margin width.^7^^,^^8^ The role of adjuvant therapies in PT treatment is not clear.^2^ Many researchers have focused on the precise effect of Radiotherapy (RT).^2^ Barth et al. reported better local control in borderline and malignant Phyllodes Tumors (PTs) with postoperative RT.^9^

These tumors have great clinical importance because, when they are not properly treated, they may have unfavorable evolution and prognosis. Given its infrequency, epidemiological and therapeutic data are scarce in the literature. The information regarding the best type of surgery, the resection margin width, and the need for and benefit of adjuvant therapies are conflicting.

## Methods

For the literature review, the MEDLINE (PubMed) databases were searched. The search strategy was based on the following terms: “phyllodes tumor” OR “phyllodes tumor of the breast” OR “borderline phyllodes tumor” OR “malignant phyllodes tumor”. Key publications were selected without date limitation and all articles in English or Portuguese were included for analysis. Titles unrelated to the topic were excluded before the screening. All studies addressing the topic of the management of borderline and malignant phyllode tumors of the breast were evaluated. This comprehensive search strategy was conducted to minimize the risk of missing relevant literature.

### *Epidemiology*

The phyllodes tumor was first described in 1774 as a type of giant fibroadenoma,^3^ and in 1838, it was named “cystosarcoma phyllodes” by the German Johannes Müller.^1^, ^2^, ^3^^,^^10^ In 1982, the World Health Organization (WHO) adopted the terminology, designating it as a phyllodes tumor, and in 2003 classified it as benign, borderline, and malignant.^1^^,^^3^ The word “phyllodes”, of Latin (phyllodium) and Greek (phullodes) origin, means “leaf-like”, a pattern that describes the histological appearance of this tumor.^11^ It was initially thought to be a benign lesion,^12^ but in 1867, Virchow characterized this tumor as having limited malignancy, capable of metastasizing.^13^ In 1943, Cooper and Ackerman confirmed the malignant potential of these neoplasms.^14^

Phyllodes tumors represent 0.3 % to 1.0 % of all primary breast neoplasms^2^^,^^15^^,^^16^ and 2 % to 3 % of fibroepithelial lesions in Western countries,^2^^,^^4^^,^^17^^,^^18^ with an estimated annual incidence of 2.1 cases per million women.^3^^,^^10^^,^^12^^,^^19^ They can occur at any age,^20^ affecting patients from the prepubertal range to the elderly.^13^ However, they are most frequent in women aged 35 to 55,^1^^,^^2^^,^^5^^,^^13^^,^^15^^,^^17^ with a peak incidence between 40- and 50-years[21,22] and an average age at diagnosis of 45-years.^23^ They are very rare in men, with only a few reported cases in the literature.^24^ Latina, Hispanic, and Asian women have a higher incidence of the disease.^11^^,^^17^^,^^25^, ^26^, ^27^ The age distribution follows an ethnic preference pattern, with Asian women being diagnosed with phyllodes tumors at a younger age compared to Caucasians.^20^ Sun et al. described two cases of phyllodes tumor in 30-year-old Chinese women.^28^ Some studies also show distinct tumor characteristics related to race. Compared to whites, Hispanics, Asians, and blacks were more likely to have large tumors.^23^ Previous publications have reported that the pediatric population is more likely to develop Malignant Phyllodes Tumor (MPT) compared to adults.^29^

### *Pathophysiology*

Its etiology has not yet been elucidated, and there are no known predisposing agents.^1^^,^^3^ Genetic risk factors for phyllodes tumors are unknown, but the literature describes it in patients with Li-Fraumeni syndrome and in mother-daughter pairs.^16^ A retrospective study conducted in Brazil identified the presence of the somatic p.R337H mutation in 5.4 % of the patients evaluated.^30^ In a cohort of North American patients diagnosed with phyllodes tumors, the use of a multi-cancer panel identified a 7.7 % prevalence of pathogenic/likely pathogenic germline mutations.^31^

Rare cases of PT in men are often associated with gynecomastia, suggesting a role for hormonal imbalance.^16^ Researchers have postulated that the stromal induction of PTs may occur due to growth factors produced by the breast epithelium.^14^^,^^16^ Trauma, pregnancy, increased estrogenic activity, and lactation have occasionally been implicated as stimulating factors for tumor growth.^3^^,^^15^^,^^16^ Unlike breast carcinoma, phyllodes tumor originates from the intralobular and periductal stroma of the breast, probably in the terminal duct lobular unit.^14^^,^^16^^,^^20^^,^^21^^,^^32^ It belongs to the group of fibroepithelial lesions, which include a combination of epithelial and stromal components.^10^^,^^21^^,^^28^ The presence of these two components is necessary to confirm the diagnosis of this neoplasm, as in the absence of the epithelial component, the diagnosis is breast sarcoma.^10^^,^^13^ From a histopathological perspective, the epithelial portion is benign, and the stromal elements, composed of hyperproliferative fibroblasts arranged in abnormal patterns (mesh, spiral, or woven), are the neoplastic components of the tumor.^2^^,^^10^^,^^17^ These are also key in differentiating between types of fibroepithelial lesions (fibroadenomas and PT), distinguishing between PT subtypes, and determining the pathological behavior of the disease.^10^^,^^33^ Interactions that occur between the epithelium and stroma seem to contribute to its pathogenesis.^32^^,^^34^ In the progression to malignancy, the stromal proliferation appears to become independent of the Wnt pathway and, presumably, of the epithelial component of these tumors.^32^^,^^35^

Histologically, PT is characterized by expanded hypercellular stroma with an increased intracanalicular growth pattern, exhibiting markedly distinct architecture with the formation of leaf-like structures in irregular, elongated clefts lined by a double layer of epithelium projecting into cystic spaces. This epithelial component consists of well-differentiated luminal epithelium surrounded by a layer of myoepithelial cells. Pseudoangiomatous stromal hyperplasia, apocrine or squamous metaplasia, usual ductal hyperplasia, and giant stromal cells can be found.^3^^,^^36^

The coexistence of PT with breast carcinoma is a rare event. These tumors can be harbored in two ways: as a distinct lesion in the ipsilateral or contralateral breast, or within the phyllodes tumor.^5^^,^^28^ Different types of carcinomas have been reported in the literature in association with PT, including ductal carcinoma in situ, tubular carcinoma, invasive ductal and lobular carcinoma, and squamous cell carcinoma.^3^^,^^28^ The detection of synchronous carcinoma is relevant, as this may indicate the need for lymph node evaluation and adjuvant therapies. Treatment should be guided by the type and staging of the diagnosed carcinoma.^28^

Macroscopically, the phyllodes tumor forms prominent, firm, and circumscribed masses, which may be surrounded by a pseudo capsule (dense compressed breast tissue containing microscopic projections of the lesion).^1^^,^^3^^,^^10^ The cut surface is grayish-white, brownish, or pink, with a homogeneous appearance or with curved clefts resembling leaf buds. Myxoid areas, cystic spaces, hemorrhage zones, and necrosis may be found.^1^^,^^3^^,^^16^

### *Diagnosis*

In 1982, the WHO established histological criteria for the diagnosis and grading of Phyllodes Tumors (PTs), which were updated in subsequent years. They classified them into three subtypes or grades: benign, borderline (also known as low-grade malignant), and malignant (or high-grade malignant).^1^^,^^3^^,^^11^^,^^22^ This classification is based on a combined assessment of five characteristics: stromal cellularity, mitotic activity, pleomorphism (stromal cell atypia), stromal overgrowth, and tumor margins^3^^,^^11^^,^^26^^,^^37^ (Table 1).

**Table 1:** Grading criteria of the World Health Organization for phyllodes tumors.

**Feature**	**Benign**	**Borderline**	**Malignant**
Tumor Borders	Well-defined	Well-defined or infiltrative	Infiltrative
Stromal Cellularity	Low	Moderate	High
Stromal Pleomorphism (Atypia)	Absent or low	Low or moderate	High
Mitotic Activity (per 10 HPF)	0–4	5–9	≥10
Stromal Overgrowth	Absent	Absent or focal	Frequently present
Malignant Heterologous Component	Absent	Absent	May be present

HPF, High-Power Field.

**Fig. 1:**
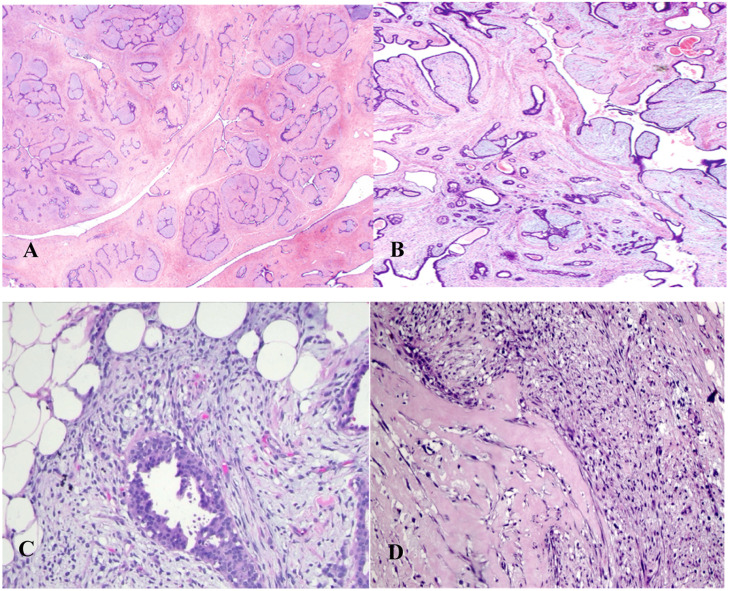
Histological Comparison of Fibroadenoma and Phyllodes Tumor Subtypes: Fibroadenoma (A), Benign Phyllodes Tumor (B), Borderline Phyllodes Tumor (C), and Malignant Phyllodes Tumor (D).

In benign phyllodes tumors (Fig. 1B), the stroma is generally more cellular than in fibroadenomas (Fig. 1A).^16^^,^^21^ Stromal cellularity may be higher in the zone immediately adjacent to the epithelium, often referred to as “periepithelial or subepithelial accentuation of stromal cellularity”. Areas of sparse stromal cellularity, hyalinization, or myxoid changes are not uncommon, reflecting stromal heterogeneity. They also present low or absent pleomorphism (the nuclei of the spindle stromal cells are monomorphic), absence of stromal overgrowth, well-circumscribed borders, and rare mitoses. Although the margins are generally well-defined, very small tumor buds can project into the surrounding tissue and, if not excised during surgery, may cause local recurrence.^1^^,^^3^^,^^16^^,^^21^^,^^38^

In contrast, a malignant phyllodes tumor (Fig. 1D) is diagnosed when it presents a combination of the following characteristics: marked stromal cellularity and pleomorphism, infiltrative borders, high mitotic activity (≥10 mitoses in 10 high-power fields of 0.5 mm^2^), and stromal overgrowth (defined as the absence of epithelial elements in one low-power microscopic field; magnification 40×; objective 4×; ocular 10×).^2^^,^^16^^,^^18^^,^^21^^,^^25^^,^^37^^,^^39^

Malignant phyllodes tumors are also diagnosed when malignant heterologous elements are present, even in the absence of other histopathological criteria.^15^^,^^36^ The finding of these heterologous mesenchymal elements is uncommon and may present as foci of fibrosarcoma, leiomyosarcoma, osteosarcoma, angiosarcoma, chondrosarcoma, and rhabdomyosarcoma.^40^

Although liposarcoma has traditionally been considered a malignant heterologous component, there is evidence suggesting a low risk of metastasis when well-differentiated liposarcomas occur as the sole heterologous element in a phyllodes tumor.^38^ These abnormal adipocytes do not exhibit amplifications in the MDM2 or CDK4 genes, in contrast to well-differentiated extramammary liposarcomas. Thus, it is recommended that the diagnosis of malignant PT not be made based solely on the finding of this heterologous component, but also on stromal characteristics.^38^

The diagnostic criteria for benign and malignant PTs are relatively well-defined; however, for borderline phyllodes tumors (Fig. 1C), they are less clear. When some, but not all, adverse histological features are found, the diagnosis of borderline PT is made.^16^

Although most authors use the WHO classification for phyllode tumors, many publications continue to classify them as low, intermediate, or high grade.^41^

The most common histological subtype is benign, with reported incidences in the literature ranging from 60 % to 78 % of cases.^3^^,^^7^^,^^16^^,^^17^^,^^23^^,^^28^ Borderline phyllodes tumors account for 6 % to 35 %, while malignant neoplasms comprise 5 % to 35 % of lesions.^3^^,^^7^^,^^15^^,^^23^^,^^28^

Phyllodes tumors typically present as a palpable, single, firm, rounded, or macro lobulated, mobile, painless breast mass with continuous and rapid growth, potentially reaching large dimensions.^1^^,^^3^^,^^10^^,^^16^^,^^22^^,^^40^^,^^41^ In some cases, the tumor may remain stable for years before accelerating in growth.^5^^,^^10^^,^^14^ Literature shows average sizes of phyllodes tumors ranging from 1.9 to 10.9 cm, with reports of lesions up to 45.0 cm.^11^^,^^16^^,^^22^^,^^23^ While some authors do not find a significant relationship between size and tumor grade,^11^ malignant phyllodes tumors often reach larger dimensions than other histological subtypes.^23^ The neoplasm can arise in any region of the breast, including the nipple.^27^^,^^42^

Due to its rapid clinical evolution, there can be distortion of breast anatomy with bulging, skin distension and ulceration, dilation of dermal vessels (varicose veins), nipple changes, and reactive axillary lymphadenopathy (due to tumor necrosis or local infection).^5^^,^^16^ Skin ulceration does not imply malignancy, as borderline and benign tumors can cause skin ischemia and secondary erosion.^36^ Bloody nipple discharge may be associated with tumor infarction.^16^ Multifocal and bilateral lesions (synchronous or metachronous) have been reported but are uncommon.^3^^,^^11^^,^^27^^,^^43^, ^44^, ^45^

Since the stromal portion represents the neoplastic component of phyllodes tumors, the World Health Organization suggests that malignant phyllodes tumors can be staged using the same criteria as soft tissue sarcomas, employing the TNM classification (classification of malignant tumors), 8th edition by the AJCC (American Joint Committee on Cancer),^10^^,^^46^ shown in Table 2.

**Table 2:** Staging system for soft tissue sarcomas (AJCC 8th edition).

**TNM Classification**	**Description**
T1	Tumor ≤5.0 cm
T2	Tumor >5.0 cm
T3	Tumor >10.0 cm and ≤15.0 cm
T4	Tumor >15.0 cm
N0	No regional lymph node metastasis or unknown nodal status
N1	Regional lymph node metastasis
M0	No distant metastasis
M1	Distant metastasis
**Stage**	**Description**
IA	T1; N0; M0; G1
IB	T2, T3, T4; N0; M0; G1
II	T1; N0; M0; G2/3
IIIA	T2; N0; M0; G2/3
IIIB	T3, T4; N0; M0; G2/3
IV	Any T; N1; M0; any G
	Any T; any N; M1; any G^a^

Data extracted from: Cates, JMM. The AJCC 8th Edition Staging System for Soft Tissue Sarcoma of the Extremities or Trunk: A Cohort Study of the SEER Database. J Natl Compr Canc Netw 2018;16(2):144–152.

a Tumor grade.

Phyllodes tumors do not have pathognomonic imaging characteristics that distinguish them from fibroadenomas or differentiate between benign, borderline, and malignant subtypes.^3^^,^^10^^,^^11^ On mammography, they typically appear as dense, round, or oval, lobulated masses with partially indistinct or well-circumscribed margins.^16^^,^^33^^,^^36^^,^^40^ Calcifications within the lesion are uncommon.^16^^,^^40^

Ultrasonographic features of these tumors include a solid, hypoechoic lesion with partially indistinct or well-circumscribed margins, usually with posterior acoustic enhancement.^11^^,^^16^^,^^47^^,^^48^ Doppler ultrasound often shows increased vascularization.^16^^,^^47^ Occasionally, the neoplasm may display cystic areas, more common in the malignant subtype.^11^^,^^16^^,^^48^ The use of elastography for differentiating between fibroepithelial lesions appears to offer promising results.^49^

On MRI, phyllodes tumors commonly present as oval, round, or lobulated nodules with well-circumscribed margins and low signal intensity on T1-weighted images, although hyperintense hemorrhagic areas may be observed. On T2-weighted images, there is often a heterogeneously hyperintense signal with slit-like spaces. Dynamic contrast-enhanced images typically show slow initial enhancement with persistent and progressive late-phase enhancement.^16^^,^^27^^,^^36^^,^^50^

Phyllodes tumors are structurally and radiologically like fibroadenomas and can easily be mistaken for them during clinical and diagnostic imaging examinations.^5^^,^^20^^,^^22^^,^^37^ However, phyllodes tumors classically reach larger volumes, potentially occupying the entire breast.^16^^,^^22^ A radiological lesion suggestive of a fibroadenoma with large dimensions should raise suspicion for a phyllodes tumor.^22^ Additionally, recent studies show that tumors larger than 3.0 cm, with irregular and micro lobulated margins, heterogeneity, hypervascularization, signal intensity less than or equal to normal fibroglandular tissue on T2, and internal cystic spaces increase suspicion for a phyllodes tumor.^3^^,^^11^^,^^36^^,^^51^^,^^52^ Furthermore, a large, irregular lesion with cystic areas is more likely to be of the borderline or malignant subtype.^11^^,^^36^^,^^40^

Imaging studies and percutaneous biopsy form the basis for the preoperative diagnosis of these lesions.^11^^,^^20^^,^^36^^,^^47^ Fine-Needle Aspiration (FNA) is usually insufficient, with a high rate of false-negative results.^5^^,^^53^ Therefore, Core Needle Biopsy (CNB) plays a valuable role, being the standard of care before surgical intervention.^5^^,^^33^ Although often diagnostic and more reliable, CNB has a false-negative rate of 25 % to 30 % and an underestimation rate of 18.8 % to 37.5 %.^37^^,^^41^^,^^54^

Despite the WHO establishing criteria for classifying phyllodes tumors, applying these criteria remains challenging.^37^ The difficulty in preoperative diagnosis of these lesions arises from several reasons: intratumoral heterogeneity; lack of well-defined histopathological parameters that accurately differentiate fibroepithelial tumors; and the mixture and overlap of histological features among phyllodes tumor subtypes, leading to significant variability in interpretation among pathologists.^11^^,^^37^ Sometimes, the biopsy fails to define the diagnosis, revealing necrotic tissue, inflammatory cells, and spindle cells with uncertain malignant potential.^20^

Due to the difficulty in obtaining a definitive diagnosis of phyllodes tumors from percutaneous biopsies, it has been suggested that the histopathological finding in this situation be described as “fibroepithelial lesion with increased stromal cellularity”, with a recommendation for complete excision of the lesion.^3^ Definitively, the diagnosis is only made after evaluating the entire surgically resected neoplasm.^20^ Differential diagnoses include cellular fibroadenoma, juvenile fibroadenoma, metaplastic carcinoma, spindle cell carcinoma, primary breast sarcomas, malignant periductal stromal tumor, and metastases from other primary tumors.^16^^,^^20^

Despite the similar clinical, radiological, and histopathological appearance between phyllodes tumors and fibroadenomas,^20^^,^^36^^,^^37^ these lesions can have drastically different clinical courses, requiring distinct therapeutic approaches.^37^^,^^55^ Therefore, accurate preoperative identification is essential, both for defining the management strategy and for better planning the surgical approach when necessary.^20^ This can avoid overtreatment, which compromises breast aesthetics, or surgical excision with inadequate margins, necessitating additional surgical procedures.^20^ Immunohistochemical markers have been suggested to increase the accuracy in distinguishing between fibroepithelial tumors, but histology remains the reference standard.^37^

Despite their rarity, phyllodes tumors are clinically important due to their unpredictable biological behavior.^32^^,^^56^ They comprise a spectrum of neoplasms that vary from benign to malignant.^16^^,^^36^ On one end of the spectrum, they behave like fibroadenomas, while on the other end, they exhibit characteristics of sarcomas, evolving aggressively.^32^^,^^36^ Although the vast majority exhibit a benign course, all phyllodes tumors have a propensity for local recurrence and can progress to malignant transformation with the risk of distant metastases.^40^^,^^41^^,^^43^^,^^57^ The genetic characteristics, histopathology, and cellular behavior of the tumor shape the extent of aggressiveness, pathogenicity, oncologic potential, and consequently, their clinical course.^36^

### *Treatment*

Complete surgical excision of the lesion, with negative margins of at least 1.0 cm, is the current cornerstone of treatment for Phyllodes Tumors (PT), regardless of histological subtype.^3^^,^^9^^,^^16^^,^^17^^,^^22^^,^^27^^,^^58^ This is the recommendation of many authors and the National Comprehensive Cancer Network (NCCN).^3^^,^^9^^,^^17^^,^^22^^,^^27^^,^^58^ However, due to the rarity of these lesions, their management has been based on data from small retrospective studies.^4^^,^^9^^,^^10^^,^^12^^,^^59^

Until the late 1970s, simple mastectomy was the standard surgical approach for all cases of PT, regardless of grade or tumor size, due to the tumor's inherently aggressive local nature.^11^^,^^17^ This aimed to ensure adequate local control and a high rate of disease-free survival.^17^ However, since then, the ideal extent of surgical resection and clear margins has been a topic of discussion.^7^^,^^8^^,^^26^

Haberer et al. emphasized the importance of wide local resection for controlling borderline and malignant phyllodes tumors regarding recurrence.^60^ Ben Hassouna et al. proposed mastectomy as the preferred approach for the malignant variety.^61^ Kapiris et al. found no statistical difference between wide local resection and mastectomy for malignant phyllodes tumors and highlighted the importance of negative surgical margins in controlling local recurrence and metastasis.^6^ A retrospective cohort with 10-years of follow-up published by Onkendi et al. in 2014, evaluating surgical resections (conservative surgery with margins < 1.0 cm, ≥ 1.0 cm, or mastectomy) in malignant and borderline PT, showed that wide tumor excision did not impact lower local recurrence.^62^ A more conservative approach, when possible, has been adopted and has shown benefits comparable to radical surgery.^10^^,^^11^^,^^19^^,^^36^^,^^43^

The main factors determining the choice of surgery type are tumor size and breast volume, aiming for adequate oncological surgery with an acceptable aesthetic outcome.^17^^,^^28^ Thus, mastectomy with breast reconstruction is often the technique of choice for large tumors (> 5.0 cm) or in small-volume breasts where the tumor-to-breast ratio is unfavorable.^11^^,^^12^^,^^36^ In other cases, Conservative Surgery (CS) with negative margins is feasible and considered the first option.^11^ Although some studies show a higher incidence of local recurrence after conservative surgery, the literature shows comparable benefits in cause-specific survival, metastasis-free survival, and overall survival compared to mastectomy.^16^^,^^19^^,^^41^^,^^43^^,^^48^ Conservative surgery with negative margins can achieve a local control rate of up to 90 %.^28^

The minimum extent of surgical margins in these tumors remains a topic of debate and controversy.^9^^,^^26^^,^^63^ Most guidelines recommend obtaining a margin of at least 1.0 cm to reduce the risk of local recurrence.^3^^,^^9^^,^^17^^,^^22^ However, this guidance, based on data from old studies with small sample sizes, is debatable and often impractical, even with mastectomy, for some large tumors.^26^^,^^33^^,^^43^ Although some authors continue to recommend wide margins, arguing that this approach is often curative and optimal for reducing local recurrences, there is no consensus on this guidance.^7^^,^^8^^,^^26^ Several studies have shown that disease-free margins are necessary and play an important role in local control and long-term survival improvement, without impacting disease-free survival and overall survival in cases with wide margins (≥ 1.0 cm).^9^^,^^12^^,^^27^

Some authors reported that surgical margins of different sizes, including “tumor not touching the ink”,^59^ ≥ 1.0 mm,^64^ and ≥ 5.0 mm,^64^ did not show evidence of harm to local recurrence and disease-free survival;[22,26,63] this raises the question that wide margins could be associated with overtreatment.^65^ In a contemporary cohort, Rosenberger et al. evaluated 550 patients diagnosed with phyllodes tumors. Over a median follow-up of 36-months, the local recurrence rate was 3.3 %, and it was not influenced by different margin widths (< 2 mm or ≥ 2 mm), nor by final margin status (positive or negative).^66^ Despite the frequent discussion about “margin extent”, the need to obtain disease-free margins is a consensus in the literature, with negative margins associated with relatively high survival rates.^8^^,^^26^^,^^43^

Although most guidelines do not specify the type of surgical treatment for different phyllodes tumor subtypes, the 1.0 cm surgical margin has been questioned, especially for benign tumors.^7^^,^^8^^,^^19^^,^^22^ Given the diversity of biological behavior and clinical evolution among PT subtypes,^10^^,^^13^^,^^25^ many authors hypothesize that different management strategies may be considered for various grades of the neoplasm.^4^ Recent studies have shown similar local recurrence rates in benign PT treated with wide surgical margins and those with margins smaller than 1.0 cm.^4^^,^^8^^,^^19^^,^^22^^,^^67^ Therefore, conservative surgery has been the treatment of choice for this subtype.^4^^,^^8^^,^^19^^,^^22^ For borderline and malignant neoplasms, most researchers agree that due to the higher recurrence risk and greater aggressiveness, a second surgical approach to obtain disease-free margins is necessary if the first surgery results in compromised margins.^22^^,^^26^^,^^36^^,^^43^ In this situation, one can opt for margin extension surgery or mastectomy.^22^ According to Neron et al., re-excision to provide negative margins improves local recurrence-free survival, metastasis-free survival, and overall survival, similar to cases of initial surgery with adequate margins.^12^ However, enlargement in cases of close but not compromised margins has no consensus in the literature.^12^^,^^28^ For recurrent phyllodes tumors, some researchers recommend a mastectomy, while NCCN guidelines suggest a new wide local excision.^36^^,^^42^^,^^58^

Since the primary route of spread for these tumors is hematogenous, lymph node involvement is rare.^10^, ^11^, ^12^^,^^17^ The literature describes a lymph node metastasis rate ranging from less than 1.0 % to 3.8 % of cases.^10^^,^^17^ Therefore, most authors suggest that axillary lymph node dissection is unnecessary and should not be routinely performed.^3^^,^^10^^,^^17^ Studies have shown that axillary management does not provide additional benefits in recurrence or survival.^36^ Although reactive lymphadenopathy is relatively common (10 % to 15 % of malignant PT cases, according to Papas et al.),^36^ with rare cases of neoplastic involvement of these lymph nodes, the literature reports that if there is clinical suspicion, the lymph nodes can be resected.^10^^,^^17^ However, some researchers recommend lymphadenectomy only when there is pathological evidence of lymph node disease.^25^ Data regarding sentinel lymph node biopsy in this type of tumor are scarce.^12^

### *Prognostic factors*

Phyllodes Tumors (PT) are known to be locally aggressive, with an inherent potential for Local Recurrence (LR), regardless of the lesion subtype, even after adequate surgical treatment.^13^^,^^33^^,^^56^^,^^63^ The literature reveals that the risk of recurrence is directly related to its histological grade.^7^^,^^68^ According to the WHO, LR occurs at an overall rate of 21 %, varying from 10 %‒17 % in benign subtypes, 14 %‒25 % in borderline, and 23 %‒30 % in malignant tumors, whereas other studies with smaller sample sizes and shorter follow-up periods report lower recurrence rates ranging from 3.3 % to 8.9 %.^3^^,^^4^^,^^8^^,^^55^^,^^66^ A literature review confirmed this information, showing that LR occurs more frequently in malignant groups (28 %) than in non-malignant ones (15 %‒17 %).^4^ Other authors have reported similar LR occurrence between borderline and malignant subtypes, with rates ranging from 21 %‒36 %.^18^^,^^33^^,^^43^^,^^48^^,^^56^ Studies show genomic similarity between these two grades, which justifies the similar aggressiveness and higher recurrence risk of these lesions.^4^ LR usually occurs within 2‒3-years of tumor diagnosis.^16^^,^^28^^,^^64^ The time to recurrence appears to be related to the degree of histological differentiation.^7^

Recurrence of phyllodes tumors can present with a more aggressive phenotype than the primary tumor in up to 30 % of cases.^26^^,^^32^^,^^56^ This tumor evolution can be justified by the heterogeneity of these lesions.^26^ A nomogram was proposed for patients with phyllode tumors to estimate recurrence-free survival. However, this tool uses only some of the histological characteristics of tumor categorization, reflecting the limitations of this grading system. Although validated, it does not distinguish LR from distant metastases.^26^

The high rate of LR is the most important prognostic factor for these tumors and thus a major concern among specialists.^48^ Consequently, possible risk factors for its occurrence have been investigated, aiming to establish strategies to reduce it.^6^^,^^68^ Several predictors of recurrence have been suggested, including tumor subtype, with a higher incidence in borderline and especially malignant neoplasms, as well as intrinsic histological characteristics such as stromal overgrowth, ≥ 10 mitoses per 10 High Power Fields (HPF), stromal pleomorphism, and tumor borders.^26^^,^^64^^,^^68^ The state of the surgical margin is widely accepted as a strong predictor of LR, with a fourfold increased risk of recurrence in cases of margin involvement by the disease.^4^^,^^10^^,^^41^^,^^43^^,^^63^ Besides the already described factors, tumor necrosis.^25^^,^^43^^,^^68^ conservative surgery,^4^^,^^12^^,^^16^^,^^68^ tumors >10.0 cm,^6^ young age,^26^^,^^43^ and fibroproliferation (coexistent fibroadenoma or fibroadenomatoid changes in the surrounding breast tissue)[25] have been variably reported as predictors for LR.

In 2023, Turashvili et al. proposed refined diagnostic criteria aimed at predicting progression to metastasis in tumors classified as malignant. The proposed diagnostic criteria were: 1) Stromal overgrowth combined with ≥1 feature(s) (marked cellularity, marked atypia, or ≥10 mitoses per 10 HPF), or 2) In the absence of stromal overgrowth, marked cellularity combined with ≥1 feature(s) (permeative borders, marked atypia, or ≥10 mitoses per 10 HPF).^69^ These criteria were later validated in a subsequent publication, demonstrating a 30 % association with progression to metastasis in the malignant phyllodes tumor group.^70^

Among all the factors presented in the literature, the state of the surgical margin has been the most consistent and reliable.^4^^,^^10^^,^^26^^,^^43^^,^^63^ In Pandey et al.'s study, all patients with recurrence had compromised margins.^71^ However, studies have shown a lack of correlation between margin width and recurrence.^63^ Thus, although a compromised margin is associated with LR, a wide margin may not provide additional benefit.^63^ In benign phyllodes tumors, the margin status seems less relevant regarding LR risk.^22^ Unfortunately, even with larger resection margins, malignant and borderline tumors present high LR rates, ranging from 21 %‒36 %.^18^^,^^33^^,^^43^^,^^48^^,^^56^ Some authors describe a higher risk of LR after conservative surgery with wide excision compared to mastectomy for malignant tumors, but without evidence of impact on overall survival.^4^^,^^12^^,^^16^^,^^19^^,^^48^

Phyllode tumors have the potential for developing metastases.^11^^,^^63^^,^^72^ The occurrence of distant disease is more frequent in the malignant subtype,^17^^,^^25^^,^^59^ with rates in the literature ranging from 1.7 % to 40 %.^10^^,^^12^^,^^19^^,^^25^^,^^37^ Although some authors describe a low risk of metastatic potential in the borderline subtype,^17^^,^^34^^,^^59^ others suggest a similar rate of distant disease between borderline and malignant phyllodes tumors.^27^^,^^56^ Some publications report rare cases of metastases in histologically benign tumors.^27^^,^^28^ The primary route of metastasis embolization is hematogenous, with the most common distant disease sites including the lungs (66 %), bones (28 %), and brain (9 %).^10^^,^^16^^,^^25^^,^^72^ However, the literature presents a substantial number of reports on unusual metastasis sites such as the liver, heart, stomach, intestines, pancreas, adrenal glands, and jaw.^10^^,^^16^^,^^72^ Histopathologically, metastasis foci lack the epithelial component, consisting of malignant stromal elements.^3^ The median interval between presentation of the primary tumor and diagnosis of distant disease ranges from 13 to 60.5 months.^6^^,^^8^^,^^72^^,^^73^

Risk factors for metastatic disease have been investigated. Tumor grade and histological characteristics (marked stromal cellularity and pleomorphism, high mitotic index, and presence of malignant heterologous elements) have been described as predictive factors.^26^^,^^63^ This indicates that inherent characteristics of PTs play an important role in the development of metastases.^26^ Although stromal overgrowth is the most relevant factor for distant disease, it is still difficult to predict its occurrence.^25^^,^^28^ Additionally, tumor necrosis has also been a predictive factor for metastasis occurrence, with its presence possibly indicating the tumor's biological activity and thus an aggressive clinical course.^25^^,^^26^^,^^63^ Other factors are controversial, such as compromised surgical margins, tumor size, and local recurrence.^4^^,^^6^^,^^36^^,^^48^

### *Adjuvant therapies*

Adjuvant Radiotherapy (RT) is well established in the treatment of breast carcinomas and is a crucial factor in local disease control, also playing an important palliative role in alleviating symptoms of metastatic disease. However, due to the rarity of Phyllodes Tumors (PT), there are no large prospective clinical trials evaluating its efficacy for this type of tumor.^15^^,^^17^^,^^56^ Although various studies have described encouraging results, supporting its efficacy and oncological safety, large-scale data are still scarce, and the criteria for its indication remain debated, with no clear guidelines for its use currently available.^43^

Given that PTs are locally aggressive and associated with high rates of Local Recurrence (LR), adjuvant RT has been suggested by many researchers as a tool to improve local control of this disease and reduce recurrence rates.^12^^,^^16^^,^^17^ However, to date, the published data are conflicting.^4^^,^^10^^,^^57^ While some recent studies have shown a reduction in LR, others have failed to demonstrate any benefit in preventing recurrence.^4^^,^^10^^,^^57^ Some researchers reported that postoperative RT would improve local control over 10-years in borderline and malignant phyllodes tumors without affecting Overall Survival (OS).^18^ Barth et al. revealed that RT was effective in local control of borderline and malignant neoplasms after breast-conserving surgery, with a significantly lower number of recurrences.^9^ Despite the probable contribution of RT in controlling LR, no proven benefit in survival has been reported.^25^ Few data have discussed the relationship between LR and the incidence of metastases, and there is also no evidence of reduction in this event.^26^^,^^36^^,^^56^

Due to the lack of national and international protocols on the use of adjuvant RT in patients with PTs, the indications for its application vary in the literature.^13^^,^^43^^,^^73^ It appears to be particularly effective in reducing recurrences in borderline and malignant PTs after conservative surgery with inadequate surgical margins (compromised margins for some authors and < 1.0 cm for others) and technical inability for new surgical intervention.^11^^,^^73^^,^^74^ Some researchers recommend RT in all cases of borderline and malignant PTs treated by conservative surgery, regardless of margin status.^12^^,^^17^^,^^22^^,^^43^ This need is increasingly emphasized as even achieving negative margins, 21 %‒36 % of these patients will experience LR.^18^^,^^33^^,^^43^^,^^48^^,^^56^ Others suggest adjuvant RT for all malignant tumors, regardless of the type of surgery and surgical margins.^33^^,^^56^ An indication considered for the use of radiotherapy is a recurrent malignant or borderline disease, as suggested in the NCCN guideline.^58^ The commonly adopted radiation protocol includes a postoperative dose of 50‒60 Gy, with 2 Gy per fraction and an additional boost to the tumor bed.^41^^,^^43^ The literature shows that not only the dose but also the timing of adjuvant RT initiation plays a significant role in preventing recurrence. When performed within one month after surgery, it shows greater benefit in reducing recurrence.^13^

The neoadjuvant scenario is even rarer for this type of tumor, with few reports in the literature. A case described in 2018 involved a recurrent malignant PT treated with neoadjuvant RT, reporting significant tumor regression and suggesting that this approach could be considered to improve treatment options and outcomes.^41^ Despite controversial results and doubts about the real benefit, adjuvant RT is increasingly used to improve local control of this disease, which has an inherently high potential for recurrence.^12^^,^^17^^,^^56^

The role of chemotherapy in the treatment of PTs is even more questionable. There are no randomized clinical trials, research is limited, and considering the adjuvant scenario, the few publications have not shown clinical benefit or impact on OS.^12^^,^^27^^,^^56^ In one study by Morales-Vásquez et al., there was no difference between receiving or not receiving chemotherapy with doxorubicin and dacarbazine in terms of survival.^39^ Some authors suggest administering systemic chemotherapy to a minority of patients, including those with large tumors, or in case of recurrence after evaluating the risks and benefits.^3^^,^^27^ However, for most researchers, chemotherapy should not be indicated except in metastatic cases.^11^ In this scenario, patients are treated according to the guidelines for soft tissue sarcoma of the NCCN and European Society for Medical Oncology (ESMO).^25^^,^^57^

The first data on the use of systemic chemotherapy were described in the 1980s and 1990s, when various regimens and combinations were used, resulting in variable responses.^17^ In the metastatic scenario, doxorubicin with ifosfamide was the most used combination, being the standard treatment in Europe.^57^ Other described regimens include liposomal doxorubicin in combination with bevacizumab;[57] epirubicin with ifosfamide;[57] cisplatin with etoposide (Etoposide);[17] in addition to chemotherapy with dacarbazine.^3^ A case report using first-line chemotherapy with doxorubicin combined with olaratumab showed rapid disease progression.^57^ In cases of disease progression, trabectedin and pazopanib are possible options.^57^

A series of 37 patients with metastatic PT was described by Mitus et al. in 2016, where most were managed with chemotherapy as the first line of treatment. The longest survival (nine months) was obtained with the combination of doxorubicin with cisplatin, cyclophosphamide, or ifosfamide, compared to endocrine therapies and single-drug chemotherapy (3 to 5 months).^8^ A second study included only seven patients, where polychemotherapy was also the main intervention, with the use of doxorubicin, ifosfamide, or cisplatin, resulting in overall poor outcomes.^75^ Alkylating agent-based regimens appear effective in metastatic malignant PTs, offering better disease control compared to those based solely on anthracyclines.^72^ Additionally, some authors described that drug combinations are not superior to single-drug chemotherapy.^72^ Numerous alterations in molecular markers of angiogenesis, such as EGFR and TP53, have been described.^72^ Thus, recent studies have developed new antiangiogenic drugs, such as bevacizumab, sunitinib, regorafenib, sorafenib, and pazopanib, which show good efficacy in sarcomas.^15^ A case report of recurrent malignant phyllodes tumor treated with RT and apatinib did not show a good response to the drug.^15^ Despite initial studies bringing divergent results, these new substances may represent an option to improve the poor outcomes currently achieved with conventional chemotherapies.

Surgical resection of metastases for palliative purposes in phyllode tumors is rarely mentioned in the literature.^3^^,^^72^ The potential benefit of this procedure has been reported for sarcomas, especially in the case of lung lesions.^72^ Therefore, in the most recent European guidelines, surgery of distant disease is recommended in the case of isolated lung metastasis in visceral and soft tissue sarcomas.^72^ A multicenter analysis by the French Sarcoma Group, published in 2019 by Neron et al. described longer survival in patients with metastatic PT who underwent lesion removal.^72^ The management and outcomes of metastatic phyllode tumors are poorly documented.^56^^,^^72^ For better results, the management of metastatic cases requires care and attention from a multidisciplinary team.^16^

The role of hormonal therapy in phyllode tumors is also questionable, without clear benefits described.^3^^,^^17^^,^^33^^,^^43^ The epithelial cells of adenocarcinoma express alpha estrogen receptors, which respond to hormonal therapy. In contrast, PTs have beta estrogen receptors in stromal cells.^17^ Besides having distinct receptors, there seems to be an indirect relationship between steroid receptors and the degree of malignancy.^36^ Although these tumors may variably present hormonal receptors in the epithelial component, hormonal therapy has not been effective in the few published studies.^17^^,^^33^ There are no randomized clinical trials on radiotherapy, chemotherapy, and hormonal therapy in the treatment of phyllode tumors. Thus, adjuvant therapy is not well-established for this type of neoplasm.^17^^,^^26^

### *Prognosis and follow-up*

Regular clinical and radiological follow-up, particularly in the initial two years post-treatment, is critical for the early detection of recurrences and metastases and is highly recommended.^28^^,^^37^ Early detection significantly influences patient management and outcomes. The aggressive nature of borderline and malignant phyllode tumors necessitates diligent monitoring and prompt intervention upon detection of recurrence or metastasis.^28^

#### General prognosis

When all subgroups of phyllodes tumors are evaluated together, they generally exhibit frequent local recurrence but have a good overall prognosis, with a 10-year survival rate of 87 %.^16^^,^^48^^,^^59^ However, when stratified by histological grade, it becomes evident that the grade is an independent predictor of survival and correlates closely with prognosis.^26^^,^^29^^,^^42^ Despite constituting the minority within this group of tumors, borderline and malignant subtypes have a much less favorable prognosis.^16^^,^^19^^,^^55^^,^^72^

#### Specific prognostic factors

The histological grade is the most significant predictor of prognosis, with borderline and malignant phyllode tumors having worse outcomes compared to benign ones.^16^^,^^19^^,^^55^^,^^72^ Larger tumor size and inadequate surgical margins are associated with poorer survival outcomes, especially in malignant phyllodes tumors.^6^^,^^43^^,^^48^^,^^62^ Table 3 summarizes the key factors influencing tumor recurrence in phyllodes tumors of the breast, highlighting the role of histological grade, surgical margins, and adjuvant therapies in recurrence risk and management.

**Table 3:** Key factors associated with tumor recurrence in phyllodes tumors of the breast.

**Aspect**	**Key-Points**
Recurrence Rates	10 %‒17 % in benign subtypes, 14 %‒25 % in borderline, and 23 %‒30 % in malignant tumors.
Time to Recurrence	Typically occurs within 2‒3-years of diagnosis.
Predictors of Recurrence	Positive surgical margins.
	Tumor size (>10 cm).
	Tumor subtype (higher in borderline and malignant).
	Histological features: stromal overgrowth, ≥10 mitoses/10 HPF, stromal pleomorphism, and infiltrative borders.
Impact of Margins	Negative margins reduce recurrence risk.
	Margin width (e.g., ≥1 cm) debated for effectiveness in reducing recurrence.
Surgical Treatment	Conservative surgery with negative margins preferred for benign subtypes.
	Borderline/malignant subtypes may require re-excision or mastectomy if margins are compromised.
Adjuvant Therapy	Radiotherapy suggested for borderline/malignant tumors with inadequate margins or inoperable cases.
	Effectiveness in reducing recurrence still debated.
Prognostic Concerns	Recurrence may lead to more aggressive phenotypes.
	Up to 30 % of recurrent tumors are more aggressive than primary tumors.
Molecular Insights	Markers like CD117 and Ki67 linked to recurrence risk.

HPF, High-Power Field.

#### Survival rates

Mortality rates for patients with borderline and malignant phyllodes tumors range from 23 % to 32 %.^56^ The literature describes a 5-year survival rate for patients with malignant phyllodes tumors varying between 54 % and 82 %.^25^ For malignant phyllodes tumors, the 10-year survival rate can be as low as 23 %, significantly impacted by factors such as tumor size and surgical margin status.^6^^,^^36^ After the development of distant metastases, the average overall survival is around 30-months.^33^

In a series from MD Anderson, the overall survival for 101 patients with phyllodes tumors was reported to be 88 % at 5-years, 79 % at 10-years, and 62 % at 15-years. For patients with nonmalignant (benign or indeterminate) and malignant cystosarcoma phyllodes, the overall survival was 91 % and 82 %, respectively, at 5-years, and 79 % and 42 %, respectively, at 10-years.^76^

### *Perspectives*

Molecular studies have been increasingly developed to better understand the origin, etiology, and biological behavior of Phyllodes Tumors (PT), with the goal of defining a less subjective classification.^36^^,^^55^ Some authors have observed comparable clonalities between phyllodes tumors and Fibroadenomas (FA), suggesting that these lesions might share a similar origin.^55^ Similar mutations in fibroadenomas and subsequent or synchronous phyllodes tumors indicate that some phyllodes tumors may arise from fibroadenomas.^42^^,^^55^

Fibroepithelial lesions frequently harbor somatic mutations, which may justify their initial growth. Alterations in MED12 and RARA are common, while phyllode tumors acquire additional genetic abnormalities in FLNA, SETD2, KMT2D, and the TERT promoter, driving their development. Phyllodes tumors also exhibit more recurrent aberrations in cancer-associated genes such as TP53, RB1, EGFR, and IGF1R, which are likely implicated in the pathogenesis and progression of these tumors.^36^^,^^55^

Various immunohistochemical markers have been studied to distinguish types of fibroepithelial lesions, improve the diagnosis and classification of phyllode tumors, and predict their clinical outcomes.^20^^,^^32^^,^^34^^,^^37^ Research has shown that markers such as p53, Ki67, CD117, EGFR, p16, and VEGF are associated with the histological grade of phyllodes tumors, with higher expression seen in malignant subtypes.^16^^,^^32^ Additionally, p53 and Ki67 have been linked to Disease-Free Survival (DFS) and Overall Survival (OS) in some studies.^16^ The expression of CD117 was found to predict tumor recurrence in a study by Tan published in 2015.^77^ Tariq et al. reported lower OS and DFS in patients expressing CD10,^78^ while Al-Masri et al. suggested that CD10 is a predictor of metastasis.^79^

Despite the evidence presented, these markers have limited clinical value in predicting the behavior and classification of phyllode tumors. Consequently, histological characteristics remain the gold standard.^20^^,^^37^ However, ongoing research and advancements in molecular biology may eventually provide more reliable and precise tools for the diagnosis, classification, and prognosis of phyllodes tumors.

## Conclusions

Many questions about the management of phyllodes tumors remain unanswered: prognostic factors are not well identified, preoperative diagnosis is complex, there is no consensus on the surgical margin threshold, and the roles of adjuvant radiotherapy and the management of metastatic disease are unclear. To date, no large-scale prospective study has been conducted due to the low incidence of these neoplasms. Consequently, existing guidelines are based on retrospective studies, and the data are still limited. An in-depth understanding of this disease may help establish strategies to reduce recurrences and develop treatment protocols, aiming for better clinical outcomes and longer survival.

## CRediT authorship contribution statement

**Aline Rocha Donato:** Conceptualization, Data curation, Formal analysis, Methodology, Writing – original draft, Writing – review & editing. **Rodrigo Goncalves:** Conceptualization, Data curation, Formal analysis, Methodology, Writing – original draft, Writing – review & editing. **Jonathan Yugo Maesaka:** Conceptualization, Data curation, Formal analysis, Methodology, Writing – review & editing. **Fernando Nalesso Aguiar:** Conceptualization, Data curation, Formal analysis, Writing – review & editing. **José Maria Soares:** Conceptualization, Writing – review & editing. **Carlos Alberto Ruiz:** Conceptualization, Writing – review & editing. **José Roberto Filassi:** Conceptualization, Writing – review & editing. **Edmund Chada Baracat:** Conceptualization, Writing – review & editing.

## Declaration of competing interest

The authors declare no conflicts of interest.
